# Assessment of sensory impairment of the upper limb post-stroke by occupational therapists within the acute setting: A mixed methods study exploring current clinical practice

**DOI:** 10.1177/03080226231184994

**Published:** 2023-07-08

**Authors:** Danielle Byrne, Liana S. Cahill, Christopher Barr, Stacey George

**Affiliations:** 1 College of Nursing and Health Sciences, Flinders University, Adelaide, South Australia; 2 Monash Health, Melbourne, Victoria, Australia; 3 Australian Catholic University, Melbourne, Victoria, Australia; 4 The Florey Institute of Neuroscience and Mental Health, Parkville, Victoria, Australia; 5 La Trobe University, Bundoora, Melbourne, Victoria, Australia; 6 Caring Futures Institute, College of Nursing and Health Sciences, Flinders University, Adelaide, South Australia

**Keywords:** Assessment, occupational therapy, stroke, somatosensory disorders, evidence-based practice

## Abstract

**Introduction:**

Sensory impairment of the upper limb is common after stroke and negatively impacts a stroke survivor’s recovery. The acute phase is a critical time for the identification of post-stroke somatosensory impairments and occupational therapists have a key role in the acute stroke setting. Sensory assessment and treatment practices of occupational therapists working in acute stroke settings are largely unknown. This study aims to describe current clinical practice and identify the barriers and enablers for the assessment of sensory impairment in patients post stroke within acute stroke units.

**Method:**

A mixed-methods approach was utilised, with an Australian national cross-sectional online survey of occupational therapists (*n* = 85) and state-based focus groups (*n* = 2). Descriptive analyses and thematic analysis were conducted.

**Findings:**

The majority of clinicians (78%) use non-standardised measures to assess for somatosensory impairment. Three qualitative themes were identified: acute setting contextual factors, individual patient characteristics and priorities, and clinician knowledge and perceived benefits.

**Conclusion:**

Occupational therapists working within acute stroke units are aware of the importance of assessing sensory impairment of the upper limb post stroke. However, the majority use non-standardised approaches and called for a standardised quick-to-administer tool kit that is readily available in acute stroke units.

## Introduction

Internationally, stroke is a persistent leading cause of disability with significant economic costs globally for post-stroke care (Lindsay et al., 2019; Rajsic et al., 2019). Somatosensory impairment, involving altered somatic senses such as touch, temperature, pain and proprioception, is common in the acute phase following stroke. Approximately 50% of stroke survivors have a reduced ability to perceive and interpret sensations, including touch and body position, in their affected upper limb (Carey and Matyas, 2011; Doyle et al., 2010). Somatosensation is critical for effective grasp, manipulation of objects and performance of skilled movements to complete a task (Schabrun and Hillier, 2009). Somatosensory impairment is associated with poorer functional outcomes after stroke (Meyer et al., 2014), longer hospital stays (Sommerfeld and von Arbin, 2004), learned non-use (Rand, 2018) and reduced activity participation (Carey et al., 2018; Connell et al., 2014). Stroke survivors report the impact of somatosensory loss to be concerning and significant, though often neglected by healthcare professionals (Carlsson et al., 2018a).

Occupational therapists are the healthcare professionals most commonly responsible for assessing upper limb somatosensation with stroke survivors (Cahill et al., 2021). Acute stroke units are considered best practice in stroke care (Langhorne et al., 2020) and are often the setting for the first evaluation of a stroke survivors’ function by the multi-disciplinary team to inform rehabilitation interventions. Stroke survivors at this early stage often present with complex neurological impairments, such as cognitive-communication deficits, which increases the complexity of somatosensory assessment. In contrast to the assessment of motor function, accurate somatosensory assessment is reliant on the adequate attention and concentration of individuals, and their ability to verbally or non-verbally communicate the detection of, and discrimination between, tactile stimuli. The somatosensory assessment methods of occupational therapists working in acute stroke units are an under-researched area of occupational therapy practice. An exploration of current practice will provide important information regarding how therapists perceive and experience challenges in the acute setting and will provide an important baseline status for future practice change.

International stroke clinical practice guidelines exist to guide somatosensory intervention. The United Kingdom and United States guidelines highlight the importance of screening for post-stroke somatosensory loss and when indicated, using standardised measures for assessment (Intercollegiate Stroke Working Party, 2023; Winstein et al., 2016). Australian and Canadian guidelines do not provide guidance regarding the use of standardised somatosensory assessment (Stroke Foundation, 2022; Teasell et al., 2020). Standardised assessments have particular advantages over subjective somatosensory assessment, including normative values for reference and established psychometric properties (Salter et al., 2005). Though guidelines advocate the use of standardised somatosensory assessment, no guidance is provided regarding specific assessments for use. Various standardised measures exist to detect and quantify upper limb somatosensory loss (Carey et al., 2020; Stolk-Hornsveld et al., 2006; Winward et al., 2002. Historically, the assessment of somatosensation has been noted as ‘frustrating’ and ‘fatiguing’ for therapists (Winward et al., 1999) and the time-consuming nature of standardised somatosensory assessments, health system pressures and lack of resources may further contribute to this. It is uncertain whether current standardised somatosensory assessments are feasible for use in a fast-paced acute stroke unit environment.

All stroke rehabilitation needs to be monitored by reliable and valid tools to objectively evaluate outcomes (Burton et al., 2013). Standardised sensory assessment is important to determine both, the specific sensory impairment to target, and to measure the effectiveness of the intervention. However, assessment of somatosensory impairments, and subsequent treatment, is often not addressed or ineffectively addressed in clinical settings, leading to inferior outcomes for stroke survivors (Cahill et al., 2018, 2021; Pumpa et al., 2015) including motor recovery in the upper limb and be a prognostic indicator in recovery (Sullivan and Hedman, 2008).

Barriers and enablers to the use of somatosensory assessments have been investigated in settings outside of acute stroke units. Lack of knowledge and skills are barriers to the implementation of evidence-based somatosensory assessment in sub-acute and community settings (Cahill et al., 2021). Underuse of standardised outcome measures with stroke survivors (Pumpa et al., 2015) may influence a clinician’s ability to detect sensory impairment and subsequently commence treatment (Connell et al., 2014). Occupational therapists have reportedly based their decision-making in sensory assessment approaches on patient characteristics, the healthcare setting and other contextual factors and describe uncertainty, for example, not knowing how to use findings to inform interventions (Doyle et al., 2014). Therapists’ decision-making is influenced by both theoretical and clinical knowledge and relies predominantly on peers as a key source of information (Doyle et al., 2014). Therapist factors and characteristics, such as knowledge and attitudes, have been found to be an important barrier to practice change in stroke rehabilitation (Juckett et al., 2020) and require consideration.

Though somatosensory recovery has been historically overshadowed by motor rehabilitation approaches, there is growing focus on somatosensation (Carlsson et al., 2018b). Cahill et al. (2021) collected data from 87 therapists, occupational therapists and physiotherapists working in inpatient and community-based settings with stroke survivors in Australia. Doyle et al. (2014) collected data from occupational therapists working across a range of settings including acute, home, nursing home, outpatient and rehabilitation. Though previous studies have explored practice in somatosensory assessment and treatment in subacute settings, the applicability of these study findings to the acute setting is not known. The context of acute settings can differ greatly to rehabilitation settings, and this warrants exploration to inform context-specific implementation strategies for acute stroke units.

Research evidence indicates the first week to 1 month post stroke is a critical time for neural plasticity (Bernhardt et al., 2017). The acute stroke unit is an important time for the screening and assessment of somatosensation, as it has been found that that if somatosensory loss is not identified early, somatosensation may fail to be ‘flagged’ and not be a focus at subsequent stages of recovery (Cahill et al., 2021). The early identification of somatosensory impairment not only ensures a vulnerable limb is protected from injury but also enables commencement of sensory-specific rehabilitation, whether provided in an inpatient or outpatient setting. This is crucial as sensory and motor systems are closely related, with both systems necessary for accurate and precise movements and to improve overall upper limb function (Carlsson et al., 2018b).

There is a gap in knowledge related to occupational therapy current practice, therapist characteristics and attitudes, and perspectives of facilitators/barriers in terms of somatosensory assessment and treatment in the acute stroke unit setting. The aims of this study were to (i) determine current clinical practice in the assessment of somatosensory impairment post stroke in the acute setting, (ii) examine relationships between therapist characteristics and attitudes towards prioritising and providing somatosensory assessment and treatment, (iii) identify occupational therapists’ perspectives of the barriers and enablers for the assessment of sensory impairment and rehabilitation in patients post-stroke within acute care.

## Method

A convergent mixed methods approach was used for this study (Creswell and Plano Clark, 2017). The quantitative component involved a national cross-sectional online survey, using both open-ended and multiple-response questions, to address aims (i) and (ii) and the qualitative component involved two focus groups in a state-based setting to address aim (iii). Inclusion criteria was occupational therapists: with recent experience, (within the past 2 years), working in an acute stroke unit for the survey; and for the focus groups, currently working on an acute stroke unit. The approach utilised a side-by-side comparison, with the quantitative and qualitative results presented separately in the Results section, and a comparison between the findings occurring in the Discussion (Cresswell and Plano Clark, 2017). Full ethics approval was granted by Monash Health Human Research Committee (RCA-18-221L).

### Quantitative study procedure

The survey was based on literature relevant to somatosensory assessments (Carey, 1995; Pumpa et al., 2015) and expert opinion. Items on the questionnaire asked occupational therapists to (1) provide demographic information; (2) describe how often they used standardised assessments to assess sensory impairments during acute care (four-point Likert-type scale categories: Never, Sometimes, Usually, Always); (3) identify which of 16 sensory assessments they used with stroke survivors to assess their upper limb and frequency of use (five-point Likert-type scale categories: Always, Often, Occasionally, Sometimes, Never); (4) identify potential barriers to completing sensory assessment and frequency of occurrence (five-point Likert-type scale categories: Strongly agree, Somewhat Agree, Neutral/Unsure, Somewhat Disagree, Strongly Disagree). The survey was sent using SurveyMonkey® (SurveyMonkey Inc., Palo Alto, CA, USA, www.surveymonkey.com) to occupational therapists registered with the Australian Health Practitioner Regulation Agency, via Occupational Therapy Australia (OTA) and online specialist neurology interest group (neurots-shrs@lists.uq.edu.au), to capture participants nationwide. The survey was conducted in 2018 with 1 month given to enable clinicians to respond. Clinicians were invited to participate if they were currently working on acute stroke units or had done so within the past 2 years. Informed consent was implied if clinicians chose to participate in the survey after reading an information sheet.

#### Data analysis

Quantitative data from the survey retrieved from SurveyMonkey, were imported to IBM SPSS Statistics 21 software (IBM Corp. Armonk, NY, USA). Quantitative data were analysed descriptively (frequencies and percentages), relationships between categorical data were examined using chi-square tests (χ^2^), and a Kruskal–Wallis test with the continuous variable of confidence with the grade of the occupational therapist. Alpha was set to 0.05.

### Qualitative study procedure

Participants for the focus groups were Melbourne-based occupational therapists recruited from the OTA neurology interest group and from a Melbourne health network, consisting of four acute settings. A semi-structured interview guide was developed (refer to Table 1) and refined based on the quantitative findings. Two focus groups with nine occupational therapists, who all provided written informed consent, were conducted in 2018. An occupational therapist with 13 years of clinical experience facilitated each focus group, while a second author acted as co-moderator, taking notes on group interaction and non-verbal communication. Focus groups were recorded, transcribed verbatim and deidentified for analysis. All focus group participants provided written informed consent to be part of this study.

**Table 1.** Focus group interview questions.

Questions
1. What has been your experience of sensory assessment in the acute setting? 2. How do you decide what screening process you use in the acute setting? 3. What factors would you consider in using standardised assessment in the acute setting? 4. Current stroke guidelines state that it is only a weak recommendation for stroke survivors with sensory loss of the upper limb, sensory-specific training may be provided. What does this mean to you? 5. Do you feel sensory impairment impacts upon the stroke survivor’s functional recovery post stroke? 6. If you had unlimited resources what would you like to be doing to practice evidence-based assessment and intervention post stroke? 7. Are you implementing sensory rehab interventions to your patients? 7a) How confident as an occupational therapist do you feel equipped with providing sensory rehab?

Qualitative responses from the surveys were recorded on an Excel spread sheet. Shared themes or responses commonly repeated throughout were noted, reviewed and included in the results.

#### Data analysis

Data from the focus groups were analysed using the four-step method described by Green et al. (2007) to generate best qualitative evidence and assist the process of analysing themes. This involved: (1) Data immersion, where the data obtained from the focus groups were repeatedly read and re-read from the interview transcripts and recordings were listened to multiple times; (2) Coding of the data, where descriptive labels were applied to segments of the transcript to make sense of the context in which statements in the interview transcripts were made. Transcripts from the focus groups were coded independently by two coders (DB and SG). The coders met to clarify coding differences and ensure consistency. Coding also involved moving back and forward through the interview transcripts, drawing on knowledge already researched on the topic and back to the original research question to examine the data; (3) Creating categories to link the codes and draw on themes. The aim was to look for data saturation as the themes should become coherent and explicable; (4) Identification of themes, interpreting the data which has emerged from the focus group and then linking the data back to a theoretical concept relevant to the study, in this case a mixed methodological method.

Strategies were used to ensure rigour of the approach to the qualitative data (Lincoln and Guba, 1985) included credibility of the study increased by the establishment of the research team, with extensive clinical and research experience in the field of occupational therapy and neurological intervention. Dependability was supported by independent coding, reflexive conversations, journaling throughout the data collection and analysis stages of all qualitative data by two researchers (Thorne et al, 1997).

## Findings

### Quantitative results

Eighty-five participants responded to the online survey, demographics are detailed in Table 2. The majority had a bachelor’s degree/entry master’s degree (*n* = 62, 72.9%), over 3–10 years of experience in stroke (*n* = 46, 54.2%), worked in a metropolitan area (*n* = 63, 74.1%) and in the public sector (*n* = 81, 95.3%).

**Table 2.** Demographics of respondents (*n* = 85).

Characteristic	Variable (*n*)	Variable (%)
*Highest level of education*		
Bachelors degree/entry masters degree	62	72.9
Postgraduate diploma/certificate	2	2.4
Postgraduate masters	19	22.4
PhD	1	1.2
Not reported	1	1.2
*Years experience with stroke clientele*		
<1	10	11.8
1–2	11	12.9
3–5	23	27.1
6–10	23	27.1
>10	17	20.0
Not reported	1	1.2
*Grade of occupational therapist*		
1	13	15.3
2	36	42.4
3	24	28.2
4	9	10.6
Not reported	3	3.5
*Geographical area*		
Metropolitan	63	74.1
Regional	17	20.0
Rural/remote	3	3.5
Not reported	2	2.4
*Sector*		
Public	81	95.3
Private	3	3.5
Not reported	1	1.2

A range of sensory assessments were used by participants (Table 3). Of the 16 assessments presented, 15 were used in the acute setting, with light touch (finger tip/cotton wool) proprioception/kinaesthesia (limb matching, thumb up/down) and pressure being the most frequently used.

**Table 3.** Survey of reported sensory assessments post-stroke in order of frequency.

Sensory assessment tool
Light touch (finger tip/cotton wool)
Proprioception/kinaesthesia (thumb up/down)
Proprioception/kinaesthesia (limb matching)
Pressure (finger tip)
Point localisation
Temperature discrimination
Two point discrimination
Texture discrimination (3–5 textures)
Functional Tactile Object Recognition Test
No specific approaches
Wrist Position Sense Test
Tactile Discrimination Test
Vibration
Nottingham sensory Assessment
Semmes-Weinstein or WEST hand monofilaments
Manual Form Perception Test
Rivermead assessment of somatosensory performance

Standardised assessments were used to assess sensory impairments: Always – 3, Usually – 11, Sometimes – 21, Rarely – 33, Never – 13. Just over half of respondents reported having a lack of knowledge of assessing sensory impairment (*n* = 44, 51.8%). Majority of respondents considered sensory assessment as a priority (*n* = 67, 78.8%) and as part of their role (*n* = 83, 97.6%). Therapists had an awareness that there is evidence to support sensory assessment (*n* = 58, 68.2%), and that the acute setting was not too early to commence sensory rehabilitation (*n* = 80, 94.1%). Time was seen as a major barrier for completing standardised assessment of somatosensory impairment for 78.8% (*n* = 67) of participants.

Missing data was in the completed surveys; 47 participants skipped the final question asking them to identify what other means they may have used to assess somatosensory impairment.

Years of experience working with stroke patients did not have an impact on occupational therapists’ opinions as to whether assessing sensation was a priority within the acute setting (χ^2^ (16) = 10.57, *p* = 0.835). There was no statistical difference as to how many years occupational therapists had been working with stroke patients and whether they used standardised assessments within the acute setting (χ^2^ (16) = 19.86, *p* = 0.227). There was also no correlation as to what geographical area occupational therapists worked within and whether they completed standardised assessments (χ^2^ (16) = 10.57, *p* = 0.542). The Kruskal–Wallis test revealed no statistically significant difference in confidence level in recommending and prescribing sensory rehabilitation interventions, across the different grades of occupational therapists (group 1, *n* = 13: grade 1, group 2, *n* = 36: grade 2, group 3, *n* = 24; grade 3, group 4, *n* = 9; grade 4, χ^2^ (3) = 1.32, *p* = 0.724).

The open-ended responses, reported a range of approaches to sensory assessment and rehabilitation including observation of performance in functional occupational based assessments and functional retraining, and some specific approaches including SENSe therapy, an evidence-based approach to somatosensory loss after stroke (Carey et al., 2011).

### Qualitative findings

Three themes were identified in the focus group data which influenced decision-making processes as to what sensory assessments were being completed within the acute setting including: acute setting contextual factors, individual patient characteristics and priorities, clinician knowledge and perceived benefits.

#### Theme 1: Acute setting contextual factors

This theme described the organisational factors in the acute setting including time, processes and environmental considerations. Time limitations were seen to be a barrier for completing a standardised assessment of somatosensory impairment, with therapists identifying that they needed a ‘quick screen’ that could ideally be undertaken in ‘5–10 mins’. Therapists identified that the quick screens assisted them with identifying the vital things that needed to be assessed and due to their time pressures within the acute setting, they felt that if there was stronger evidence for sensory impairment interventions, this would assist in their justification of how their time is best spent. One clinician commented ‘I think as well with the time pressures and the like, quite large range that we have to cover when assessing the upper limb, that often you will not do a really in-depth assessment’.

Occupational therapists in the acute setting described themselves as ‘time poor’, needing to do their assessments ‘quickly’ and being very aware of their ‘time management’ in order to address other organisational factors such as pressures to ‘discharge patients and make quick decisions’, the case load pressures including the sheer number of patients needing to be assessed as well as needing to assess for other neurological impairment found within stroke patients, that were also perceived to be within the occupational therapist’s role for neurological assessment. One therapist stated: ‘Acute hasn’t changed . . . we are still busy and we still don’t get to it’ highlighting both the time and organisational pressures felt by acute therapists.

Therapists agreed that if a ‘*tool kit*’ was readily available to them, which delivered best evidence-based practice on what needed to be assessed in assessment of sensation, then they would be more inclined to use this, as it would save them time and provide them with ‘a little bit of guidance’.

Therapists indicated that they might have more time to spend on in-depth assessment for those patients that are not ‘walking out the door’ but may be waiting for a rehabilitation bed.

Therapists described they were likely to choose their assessments depending on what resources were readily available to them in their immediate environment. Difficulty was described in completing standardised assessments due to physical space ‘with a table at the right height, that like fits with a chair, to have a really good set up for the kind of assessment is quite a challenge’. Access to resources including standardised assessments was described as an organisational factor hindering evidence-based practice.

Interestingly, therapists reflected on the differences between acute and rehabilitation ‘I haven’t done too many standardised assessments in this setting, compared to rehab’ more using observation and ‘then looking at things in a functional context, and how’s that going to impact on discharge planning’. Furthermore, the approach to sensory assessment in a non-standardised way was described as not increasing confidence in informing practice related to sensory intervention: ‘you don’t walk away with a great deal of confidence, I don’t think in your assessment’.

#### Theme 2: Individual patient characteristics and priorities

Occupational therapists in the acute setting perceived a range of patient factors that contributed to their selection of assessment of sensation or timing of assessment. Patient factors that they identified included type of stroke, cognitive impairment/communication impairment and fatigue. It was reported that the type of stroke influenced practice in relation to sensory assessment:

I think depending on where the stroke is as well, like if it’s in an area where you’re expecting certain motor or sensory changes, you’d look at it a lot more. Whereas someone with like occipital stroke, you’re less likely to see sensation, so probably be more likely to gloss over it.

Also, the stroke severity influenced sensory assessment as those with mild stroke may only be seen once and then be discharged from occupational therapy ‘by the time you’re looking at motor, upper limb, your sensory, your cognition, your functional cognition, vision- it takes a long time to the do the education’. One therapist described the impact of patient fatigue:

Often it can be too early to complete extensive assessments as we see them within 24 hours post stroke and fatigue is often a large barrier to completing all assessments, especially when the physiotherapist, speech pathologist and doctors will then see them that same day.

Another patient factor identified was patients not realising the importance of sensation on function. One therapist stated ‘If they don’t have motor movement they’re probably not noticing it yet, not realising the potential impact sensation loss has until they start doing things’. Another therapist commented, ‘I think it’s even to do with the patient priorities as well, I don’t think they fully understand what’s gone on with their arm yet and maybe they don’t have a lot of motor return’, justifying their clinical reasoning as to why assessment of sensation may not be prioritised. Additionally, other patient factors including a reduced tolerance for participation in assessment or the prioritisation of other short-term goals such as returning to moving, walking or increasing their independence of basic activities of daily living, which are apparent in the acute setting when they are coming to terms with living with stroke ‘trying to get some normal independence in that daily routine’.

#### Theme 3: Clinician knowledge and perceived benefits

Occupational therapists mostly reported that they were completing sensory assessment in a ‘global, gross way’, screening the patient for sensory impairment, mostly using ‘clinical judgement’. Sensory screens tended to include light touch, pain, proprioception, temperature, screening for inattention using bilateral stimulus and stereognosis.

Therapists reported a lack of knowledge of evidence-based practice related to sensation, describing the need for education about sensory intervention and assessment: ‘I think it would be good to have further education as well about what standardised assessments are available we can be implementing in our practice’.

Occupational therapists described the need to complete standardised sensory assessments to inform intervention: ‘once you’ve picked up a sensory problem, then you want to start putting in the intervention straight away’ and ‘to be able to have some kind of standardised assessment so that when they go to rehab you can hand that over, and the rehab therapist is like ‘Okay I know exactly where we are at’ to facilitate early rehabilitation and continuity of care, and early education of patients and families’. Measuring change across a patient’s continuum of care was also reported as a benefit of a standardised approach to sensory assessment.

Local clinical guidelines also appeared to influence occupational therapists’ choice of assessment of sensation of the upper limb, with clinicians citing they utilised local assessment forms to guide their assessment. Clinicians also advised that stroke guidelines assisted them with knowing where to best spend their time, reiterating the theme that time is an essential factor for acute clinicians ‘you’ve got motor evidence for motor retraining that’s strong recommendations than you’ve got a weak sensory recommendation . . . maybe that’s where I will invest my time’. Another clinician commented:

I think it gets quite overshadowed by motor in acute and the priorities sitting with sub lax prevention and motor recovery rather than so much sensory, I think it’s put on the back burner and said, ‘Oh they might cover that off in rehab, we’ll leave that for now.

## Discussion

The key finding of this study from the national survey of current clinical practice in Australia is that the majority of occupational therapists in acute stroke settings use non-standardised assessments of somatosensory impairment with stroke survivors. Benefits of standardised assessment approaches were acknowledged by therapists, including the accurate detection of sensory deficits to promote early rehabilitation and as outcome measures for use across the rehabilitation continuum. Furthermore, the use of non-standardised approaches were described as not increasing confidence of occupational therapists in their practice. There is an evidence practice gap between international clinical guidelines, in terms of using standardised approaches, and current clinical practice in somatosensory rehabilitation in acute settings (Intercollegiate Stroke Working Party, 2023; Winstein et al., 2016).

In terms of the second aim of this study of examining the relationship between therapist characteristics (years of experience, location) and attitudes towards prioritising and providing somatosensory assessment and treatment, no relationships were found, as the majority of therapists viewed sensory assessment as a priority and a significant role for occupational therapists working in acute stroke units. In terms of evidence to support the choice of sensory approaches in the acute setting, majority in this study reported knowledge of evidence, referring to local and stroke clinical practice guidelines. This awareness of evidence influenced decision-making of where to invest their time, and therapists viewed the evidence-base for motor recovery as stronger than that for sensory rehabilitation. This is consistent with literature such as Cahill et al. (2021) noting that despite somatosensory impairment in stroke survivors being prevalent clinicians often give precedence to motor recovery post stroke. Occupational therapists working in the acute setting have indicated the higher level of evidence for motor recovery interventions in current stroke guidelines determines or prioritises where their time is best spent, as current guidelines indicate a weak recommendation for somatosensory rehabilitation (Stroke Foundation, 2022).

Furthermore with therapist factors and characteristics, such as knowledge and attitudes, an important barrier to practice change in stroke rehabilitation (Juckett et al., 2020), the results of this study indicate that there was a high attitudinal support for completing sensory assessment in the acute setting, but a high proportion of therapists (53%) reported lacking knowledge in how to conduct sensory assessment. This suggests that therapists require upskilling, to increase knowledge of sensory assessment and treatment approaches in the acute setting. Quantitative data found no association between years of therapist experience, the grade of therapist or geographical area as to whether standardised sensory assessment approaches were used, suggesting that approaches to increase knowledge and confidence need to be targeted across all levels, and metropolitan and rural health organisations. Previous research provides insights into approaches including peer observation, continuing education, awareness of research and reflective practice (Doyle et al., 2013, 2014).

Unsurprisingly, time was cited as the main barrier in acute stroke units for not completing standardised sensory assessments, with organisational factors of short lengths of patient stay and discharge priorities impacting. Findings from this study are consistent with Pumpa et al. (2015) in a subacute rehabilitation setting, where non-standardised assessments were typically used due to lack of time, resources or equipment, and a lack of awareness of more recent research findings and specific interventions to implement evidence-based measures. Development of a brief, standardised tool kit that is quick to administer (5 minutes) and readily available in acute stroke units was recommended by participants to overcome organisational barriers and assist therapists to deliver best evidence-based assessment for stroke survivors. Connell et al. (2014) also identified that there was a need for evidence based and practice-appropriate clinical assessment tools related to sensory rehabilitation. This indicates a need to investigate implementation strategies for standardised approaches to sensory assessment specifically in the context of the acute setting, as context is critical for implementation success (Nilsen and Bernhardsson, 2019).

Similarly to our results, Pumpa et al. (2015) found therapists either used subjective reports or observations within functional activities, such as personal care or hot drink assessments, to assess sensation. Acute therapists cited that they would often begin by completing a ‘quick’ neurological screen, determining whether they needed to complete an in-depth assessment or assume that their sub-acute or community therapy colleagues would be better placed to conduct standardised, lengthy somatosensory assessments. Doyle et al. (2013) also noted that occupational therapists tended to complete a sensory assessment if they were concerned about safety or wanted to determine the impact of upper limb sensory loss on occupational performance.

Differences in quantitative and qualitative data were also identified. Interestingly, the survey data demonstrated that the majority of occupational therapists (72.84%) strongly disagreed that it is too early to commence sensory rehabilitation in the acute setting; however, the focus group data revealed a range of factors within the acute clinical setting that may be a barrier to commencing rehabilitation, including individual patient factors such as fatigue. Also, it was observed that sensation is not always the priority of the patient immediately after a stroke, when regaining mobility may be a more pressing concern. This differs from the literature that found stroke survivors perceived sensory retraining as rewarding and value its potential to improve their function; however, the participants in this study had participated in sensory training and also were more than 16 weeks post-stroke (Turville et al., 2019). Thus, a consideration to increase patient participation in sensory approaches in the acute setting may be education related to somatosensation and the relationship between sensation and function. Effective education is likely to require consideration of factors specific to an acute stroke setting such as time, space and resources, to enhance its practical application.

This study found that occupational therapists working in an acute setting report similar factors to healthcare professionals in rehabilitation and community settings influencing the provision of somatosensory rehabilitation after stroke (Cahill et al., 2021). In the context of rehabilitation: *individual* – (uncertain, unskilled therapist), *patient* (understanding and priorities) and *organisational* (pressures and resources) factors were identified. The findings from our study expand understanding of the unique characteristics in the acute setting: *individual –* knowledge and perceived benefit; *patient –* characteristics and priorities on function; *organisational –* set up, focus on discharge, length of stay. Tailored implementation strategies, such as a modified, brief somatosensory kit may address these factors to increase the use of evidence-based somatosensory rehabilitation in the acute setting.

Furthermore, the results support the literature (Doyle et al., 2013), which identified that the patient’s cognitive or communication impairments impacted upon the therapist’s decision to complete a sensory assessment (Doyle et al., 2013). Data from the focus groups found that therapists considered other factors including fatigue, tolerance, patient priorities, goals and maintaining a client-centred approach. Occupational therapists were torn between their own clinical knowledge, knowing how sensation will impact upon functional outcomes and prioritising perceived ‘quick wins’ to maintain motivation early in stroke recovery, relevant in the acute setting where patient flow to other facilities happens swiftly.

### Limitations

Limitations of this study include a small sample size within the focus groups, which does not meet recommended numbers (5–10), thus it is unlikely saturation was reached (Morgan and Hoffman, 2018). Furthermore, participants were Melbourne-based; therefore, rural and interstate therapists were not represented and may have had different opinions. Small numbers for the qualitative findings may influence the generalisation of the findings more broadly. Occupational therapists were also recruited from neurology special interest groups, which may have caused selection bias. However, in the nationally distributed online surveys, there did not appear to be any significant statistical differences in responses to the questions when comparing factors such as experience and geographical locations.

## Conclusion

The majority of occupational therapy clinicians are using non-standardised approaches for the assessment of somatosensory impairment post stroke in the acute setting. Occupational therapists are aware of the importance of somatosensation; however, due to the absence of strong evidence in current stroke guidelines and a strong recommendation for what standardised assessment should be utilised, they do not prioritise the assessment of somatosensory impairment in acute stroke units. The barriers identified in this study included a perceived lack of time and resources and patient factors to deliver evidence based assessment of sensory impairment with stroke survivors.

**Key findings**

- Acute occupational therapists face unique organisational challenges influencing assessment selection.
- Acute occupational therapists need a quick standardised assessment to assess for somatosensory impairment.
- Tailored implementation strategies considering the acute setting contextual factors, patient characteristics/priorities and clinician knowledge and perceived benefits are required.

**What the study has added**

This study adds new knowledge about occupational therapists approaches to sensory assessment in acute stroke units, namely consideration of knowledge and perceived benefits, and patient characteristics to embed evidence in practice.
